# Serum free light chain level at diagnosis in myeloma cast nephropathy—a multicentre study

**DOI:** 10.1038/s41408-020-0295-4

**Published:** 2020-03-03

**Authors:** Punit Yadav, Insara Jaffer Sathick, Nelson Leung, Elizabeth E. Brown, Mark Cook, Paul W. Sanders, Paul Cockwell

**Affiliations:** 1 Department of Renal Medicine, University Hospitals Birmingham NHS Foundation Trust, Birmingham, UK; 2 Institute of Inflammation and Ageing, University of Birmingham, Birmingham, UK; 3 Mayo Clinic, Rochester NY, USA; 4 Department of Pathology, University of Alabama at Birmingham, Birmingham AL, USA; 5 Department of Haematology, University Hospitals Birmingham NHS Foundation Trust, Birmingham, UK; 6 Department of Medicine, University of Alabama at Birmingham, Birmingham, AL USA; 7 Birmingham Veterans Affairs Medical Center, Birmingham, AL USA

**Keywords:** Medical research, Translational research

## Abstract

Myeloma cast nephropathy (MCN) is a common cause of severe renal impairment in multiple myeloma (MM). The level of free light chain (FLC) that causes MCN varies substantially and there is uncertainty about the threshold level that should be used to inform clinical practice. In a multicentre cohort study of 103 patients with a diagnosis of MM and biopsy-confirmed MCN made between 2002–2014, we report prospectively measured levels of serum FLC at diagnosis obtained using a single nephelometric assay (Freelite®) and we explore the relationship between serum FLC level at diagnosis with renal outcome and patient survival. Using a landmark approach, overall survival (OS) was compared between patients who achieved independence from dialysis compared to those who remained dialysis dependent at 3-month, 6-month, 9-month, and 12-month time points. The median serum FLC level at diagnosis was 7531 mg/L (range 107–114600). Serum creatinine was 535 μmol/L (range 168–2993) and eGFR 7 ml/min/1.73 m^2^ (range 1–34). Six patients (5.8%) had an FLC level <1500 mg/L, which is the International Myeloma Working Group threshold for MCN and two patients were below the International Kidney and Monoclonal Gammopathy working group threshold of 500 mg/L; one was hypercalcaemic, and one had high-normal serum calcium level and had received a non-steroidal anti-inflammatory agent. Sixty-nine (67%) patients required haemodialysis treatment of whom 36 (52.1%) recovered independent renal function. Sixty-six (64%) patients died with a median OS of 2.5 years (95% CI 1.8–3.3). A landmark analysis revealed that independence from dialysis was associated with improved survival at 3-months (*P* = 0.003), 6-months (*P* = 0.035) and 9-months (*P* = 0.014); there was no survival benefit observed beyond 12 months (*P* = 0.146). Serum FLC level at diagnosis was neither associated with renal function recovery nor with OS. This is the largest reported cohort of patients with biopsy-confirmed MCN and prospectively measured serum FLC levels. These results indicate that a serum monoclonal FLC > 500 mg/L should be considered the threshold level associated with the development of MCN.

## Introduction

Renal function impairment (RI) is reported in up to 50% of patients with multiple myeloma (MM) at presentation^1–6^. Severe RI as defined by an estimated glomerular filtration rate (eGFR) <30 ml/min/1.73 m^2^ is present in up to 20% of patients with MM at diagnosis^7^, which approximates to the level of renal dysfunction defined by the renal component of the end-organ damage (hypercalcemia [C], renal failure [R], anaemia [A], or bone lesions [B], herein referred to as CRAB) classification system^8^. The predominant cause for severe RI is the pathognomonic distal tubular lesion, myeloma cast nephropathy (MCN), which is a direct consequence of the secreted monoclonal immunoglobulin (Ig) free light chain (FLC)^9^. The level of pathogenic serum FLC that causes MCN varies substantially and there is uncertainty about the threshold level that should be used to inform clinical practice^10^.

A paradigm shift in the management of patients with MM has been the development of the serum FLC assay^11^, which is now the test recommended by the International Myeloma Working Group (IMWG) for the screening of patients with a suspected paraproteinemia^12^. The IMWG recently revised the criteria for the diagnosis of MM in cases without CRAB features to include an involved to uninvolved serum FLC ratio >100 in association with an involved FLC level above 100 mg/L, clonal bone marrow plasma cells ≥60 percent, or more than one focal bone lesion (>5 mm) identified using magnetic resonance imaging^13^.

The serum FLC assay is used in patients with unexplained acute kidney injury (AKI) to screen for potential MCN. However, the range of FLC at which MCN can occur is uncertain. The IMWG associated RI as a consequence of MCN to a serum FLC level above 1500 mg/L^13^, whereas the International Kidney and Monoclonal Gammopathy (IKMG) Research Group proposed a serum FLC level above 500 mg/L^14^. We recently analysed the Myeloma IX study and found that an FLC level above 800 mg/L had the highest sensitivity and specificity for severe RI defined by an estimated glomerular filtration rate (eGFR) <30 ml/min/1.73 m^2^ at diagnosis^15^. However that study did not include patients who required dialysis and information on renal histology was not available. Additionally, it is also uncertain if the level of FLC in serum at presentation is an independent determinant of outcome in patients with AKI and MCN.

Therefore, the aim of this study was to primarily report the range of FLC levels in serum in patients with newly diagnosed MM who had AKI from renal biopsy-confirmed MCN. Additionally, we also report renal outcomes for those on dialysis and overall survival (OS) for the entire cohort.

## Patient and methods

This was a multicentre study of newly diagnosed patients with MM and with biopsy-confirmed MCN between 2002 and 2014. Patients had presented at three tertiary centres: University Hospital Birmingham NHS Foundation Trust (UHB), Birmingham, UK; Mayo Clinic, Rochester, Minnesota, USA and; University of Alabama at Birmingham (UAB), Birmingham, Alabama, USA. We evaluated prospectively measured levels of involved FLC in serum using a single nephelometric assay (Freelite®, The Binding Site, Birmingham, UK). Informed consent was obtained from all patients. Approval for conduct of this study was obtained from the local ethics committee or the Institutional Review Board from the three centres in accordance with the Declaration of Helsinki.

**Fig. 1:**
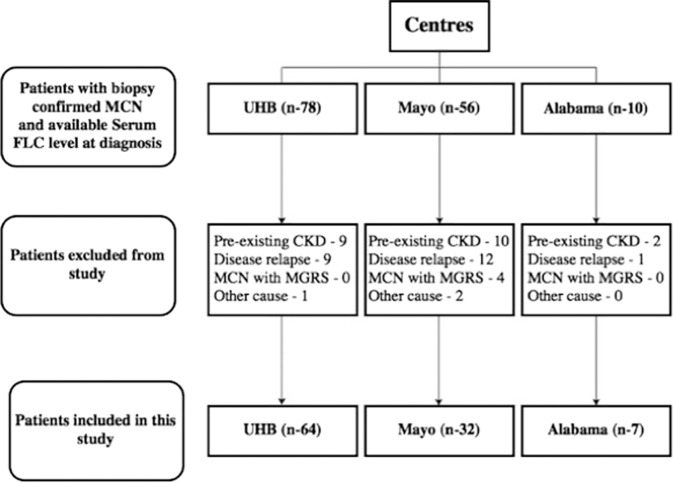
CONSORT diagram showing patients included in study. *UHB* University Hospital Birmingham, *FLC* free light chain, *MCN* myeloma cast nephropathy, *MGRS* monoclonal gammopathy of renal significance.

Figure 1 is a CONSORT diagram showing the characteristics of patients included in the study. Inclusion criteria comprised all patients with a new diagnosis of MM and biopsy proven MCN. Patients that were excluded from analysis comprised: (a) those with relapsing/remitting MM; (b) those known to have chronic kidney disease (CKD); (c) renal transplant recipients; (d) those with a histological diagnosis of MCN and other paraproteinemia e.g. amyloidosis, renal monoclonal immunoglobulin deposition diseases, or any other monoclonal gammopathy of renal significance (MGRS); and (e) patients in whom serum FLC levels were measured after commencement of anti-myeloma therapy.

Variables included in the analysis were: patient age at diagnosis, gender, ethnicity, date of presentation with RI, Modification of Diet in Renal Disease study equation (MDRD) eGFR (ml/min/1.73 m^2^), corrected serum calcium, haemoglobin, paraprotein and FLC type, serum FLC level at presentation, date of renal biopsy, days from presentation to renal biopsy, days from presentation to receipt of first dialysis, date of dialysis independence, duration of dialysis, percentage of plasma cell infiltrate in the bone marrow, OS and time to death.

Statistical analysis was performed using SPSS® for Windows, version 21.0 (SPSS Inc, Chicago, IL) and Graph Pad Prism 5.0 (GraphPad Software Inc, San Diego, CA). Categorical variables were summarised as frequencies and percentages. Comparisons for categorical variables among different groups were made with the chi-square test and Fisher’s exact test where appropriate. Continuous variables were expressed as mean and standard deviation (SD) if the data was normally distributed or median with range for non-normally distributed data. Non-parametric comparisons were performed by using the Mann-Whitney *U* test. Correlation analysis between patients presenting with eGFR <15 ml/min/1.73 m^2^ irrespective of haemodialysis status at diagnosis and serum FLC levels was performed with Spearman’s rank test. In addition to reporting levels of FLC in serum that were associated with MCN, we also examined variables collected at presentation that correlated with renal function recovery in patients on haemodialysis and OS. For patients on haemodialysis, renal function recovery was defined as a sustained independence from dialysis treatment for at least two months. Patients were followed until death from any cause or censored on 31^st^ December 2014, if they were still alive at this date, for survival analysis. In order to reduce the possibility for lead-time bias, OS for patients who achieved independence from haemodialysis and were alive were compared with OS for patients who were haemodialysis dependent and alive in a landmark analysis at 3 months, 6 months, 9 months months after presentation with a new diagnosis of MCN. Patients who died before the landmark time were excluded and OS for the landmark analysis was performed from the landmark time to death. Outcomes in patients with MM have progressively improved with time due to the advances in supportive care and introduction of novel anti-myeloma therapies. From 2008, the chemotherapy regimens based on the proteasome inhibitor bortezomib became the standard of care for MM and severe AKI, and hence differences in survival were compared between those diagnosed with MCN before and after this time-point. All statistical tests were two sided and a *P* value of <0.05 was considered as significant.

## Results

### Patient demographic and disease characteristics

103 patients with incident MM, with no previous history of CKD, serum FLC level available at diagnosis and histologically confirmed MCN by renal biopsy were included in the study. Table 1 summarises the baseline characteristics of patients in the study. The median age at presentation was 63 years (range 38–85), 57.3% were of male gender, and 88.3% were white. Light chain restricted MM (47.6%) was the predominant paraprotein type. An involved monoclonal kappa (κ) FLC isotype was present in 54.4% and an involved lambda (λ) FLC in 45.6% of patients.

**Table 1:** Patient characteristics.

	All patients (*n* = 103)	Patients not requiring dialysis (*n* = 34)	Patients requiring dialysis and recovering renal function (*n* = 36)	Patients requiring dialysis and not recovering renal function (*n* = 33)
**Centres**				
UHB	64 (62.1)	14 (41.1)	27 (75.0)	23 (69.7)
Mayo	32 (31.1)	17 (50.0)	7 (19.4)	8 (24.2)
UAB	7 (6.8)	3 (8.8)	2 (5.5)	2 (6.0)
**Age (years)**	63 (38–85)	64 (38–84)	60 (43–72)	65 (45–85)
**Gender**				
Male	59 (57.3)	19 (55.9)	25 (69.4)	15 (45.4)
Female	44 (42.7)	15 (44.1)	11 (30.5)	18 (54.5)
**Ethnicity**				
White	91 (88.3)	32 (94.1)	30 (83.3)	29 (87.8)
Non-white	12 (11.6)	2 (5.8)	6 (16.6)	4 (12.1)
**Paraprotein type**				
Light chain restricted MM	49 (47.6)	14 (41.1)	15 (41.6)	20 (60.6)
Whole Ig MM	54 (52.4)	20 (58.8)	21 (58.3)	13 (39.3)
**FLC isotype**				
Kappa (*κ*)	56 (54.4)	16 (47)	19 (52.7)	21 (63.6)
Lambda (*λ*)	47 (45.6)	18 (52.9)	17 (47.2)	12 (36.3)
**FLC level (mg/L)**				
FLC level irrespective of isotype	7531 (107–114,600)	6724 (107–32,294)	7960 (580–43,161)	10134 (1620–114,600)
* κ* FLC level	6965 (201–46,000)	6474 (201–32,294)	7844 (580–28,622)	9302 (1620–46,000)
* λ* FLC level	8220 (107–114,600)	6853 (107–20,000)	8108 (1971–43161)	12667 (1760–114,600)
**Serum creatinine (μmol/L)**	535 (168–2993)	349 (168–1141)	685 (329–2210)	778 (265–2993)
**eGFR (ml/min/1.73 m**^**2**^**)**	7 (1–34)	12 (3–34)	6 (2–15)	5 (1–17)
**Haemoglobin (g/L)**	90 (46–131)	99 (54–129)	86 (56–131)	86 (46–126)
**Corrected calcium (mmol/L)**	2.35 (1.65–4.25)	2.38 (1.96–4.25)	2.32 (1.65–3.27)	2.43 (1.97–4.23)
**Percentage plasma cell infiltrate on bone marrow**	55 (1–90)	60 (1–85)	50 (10–90)	55 (20–90)

Mean ± standard deviation or median (range) reported for continuous variables and frequency (percentage) reported for categorical variables.

*UHB* University Hospital Birmingham, *UAB* University of Alabama at Birmingham, *MM* multiple myeloma, *Ig* immunoglobulin, *FLC* free light chain.

**Fig. 2:**
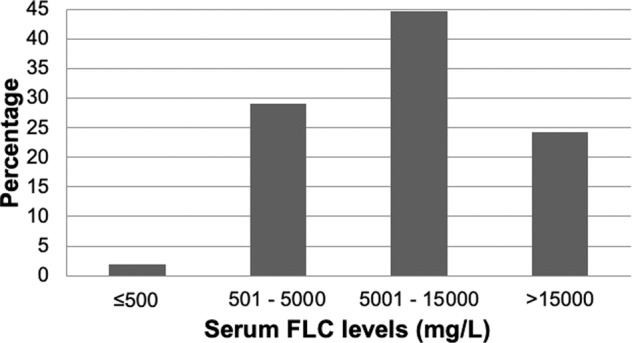
Distributions of FLC level in serum. Percentage of patients with FLC levels in serum grouped by <500 mg/L, 501–5000 mg/L, and >1500 mg/L.

The median involved serum FLC level at presentation was 7531 mg/L (range 107–114,600). Distribution of FLC levels in serum for the entire cohort is presented in Fig. 2. There was no significant difference in the levels of FLC in serum for patients presenting with involved *κ* FLC or involved *λ* FLC isotypes (*P* = 0.211). Two (1.9%) patients presented with an involved serum FLC level that was ≤500 mg/L at diagnosis; one patient was hypercalcaemic, and the other patient had a high-normal serum calcium level and was on a non-steroidal anti-inflammatory drug.

### Renal characteristics at diagnosis

**Fig. 3:**
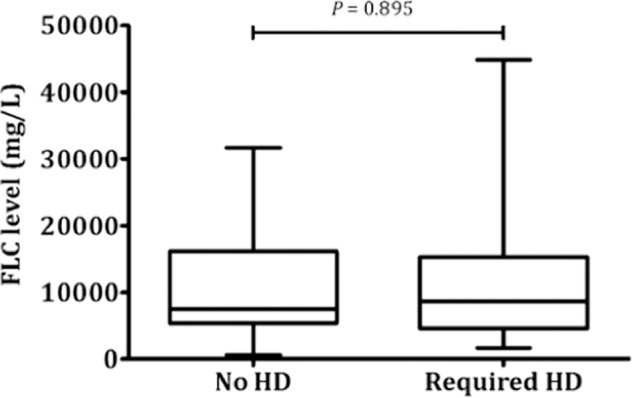
Serum FLC levels at diagnosis. Box-plot of serum FLC levels at diagnosis in patients presenting with eGFR <15 ml/min/1.73 m^2^ by dialysis status (HD, haemodialysis).

Sixty-nine (66.9%) patients required haemodialysis after the diagnosis of MCN. Of these 51 patients received extracorporeal treatment; 31 with plasma exchange (PE, Mayo Clinic and UAB) and 21 with high-cut off haemodialysis (HCO-HD, UHB). The median time from presentation to start of haemodialysis was one day (range 0–202); two patients started haemodialysis 30 days after presentation (day 49 and day 202, respectively). There was no significant difference in serum FLC levels in patients presenting with eGFR <15 ml/min/1.73 m^2^ at diagnosis irrespective of haemodialysis status (median serum FLC level in patients not requiring haemodialysis 7444 mg/L [range 575–32,294] and 8643 mg/L [range 580–114,600] for those requiring haemodialysis, *P* = 0.895) (Fig. 3).

### Renal outcomes and patient survival

Thirty-four (33%) patients had no requirement for haemodialysis treatment at diagnosis. For the 69 (66.9%) patients who required haemodialysis at diagnosis, 36 (52.1%) achieved recovery of renal function. The median duration of haemodialysis treatment was 36 days (range 10–701).

We found no significant difference in age, eGFR, corrected calcium, haemoglobin, and involved serum FLC level at presentation between patients who required haemodialysis and recovered renal function compared to those who remained dialysis-dependent. There was no difference in the renal recovery rate for patients who received PE (*P* = 0.282) or HCO-HD compared to standard dialysis (*P* = 0.336).

Patients who did not require dialysis had higher haemoglobin concentration (median 98 g/L [range 54–129] vs 86 g/L [range 46–126] respectively, *P* = 0.010), and lower involved serum FLC level (median 6724 mg/L [range 107–32,294] vs 10134 mg/L [range 1620–114,600], *P* = 0.044) compared to those who remained dialysis-dependent.

At the end of the follow-up period, 66 (64%) patients had died. The median OS from diagnosis was 2.5 years (95% CI 1.8–3.3). There was no significant difference in OS between patients who received haemodialysis at presentation (median 2.0 years; 95% CI 0.9–3.1) compared to those who had no requirement for dialysis (median 3.5 years; 95% CI 1.6–5.5) (*P* = 0.248). This finding was unchanged in an analysis that excluded the patient who started haemodialysis on day 202 after the diagnosis of MCN. Patients who received haemodialysis at diagnosis and subsequently recovered renal function showed a significantly improved OS (median 3.3 years, 95% CI 1.6–5.0) compared to those who remained dialysis-dependent (median 0.8 years, 95% CI 0.6–1.0) (*P* < 0.0001). On performing a landmark analysis, independence from dialysis at 3-month (*P* = 0.003), 6-month (*P* = 0.035) and 9-month (*P* = 0.014) time points were associated with improved survival whereas no such survival benefit was observed beyond 12 months (*P* = 0.146) (Table 2).

**Table 2:** Landmark analysis of associations between haemodialysis independence and overall survival in patients with MCN (*n* = 69).

		HD independent			HD dependent			
Landmark time	Pts excluded (*n*)	Patients (*n*)	Deaths after landmark time (*n*)	Median OS (years)	Patients (*n*)	Deaths after landmark time (*n*)	Median OS (years)	*P-*value
3 month	9	35	22	3.85	25	21	1.12	0.003
6 month	17	34	21	3.85	18	15	1.73	0.035
9 month	19	32	19	4.23	18	15	1.72	0.014
12 month	25	31	18	4.23	13	10	2.93	0.146

The median OS amongst patients who presented after 2008 was 3.8 years (95% CI 2.5–5.1) and was significantly better compared to those who presented before 2008 (median 1.7 years, 95% CI 0.6–2.9) (*P* < 0.006). We found no statistically significant change in the rates of renal function recovery (*P* = 0.151) between those presenting before or after 2008.

## Discussion

In this multicentre study the principal findings were: (1) the variable range of FLC levels in serum that were observed in patients presenting with biopsy-confirmed MCN; (2) Six patients had an FLC level <1500 mg/L, the level recommend by the IMWG for MCN; (3) two of the 103 patients presented with FLC levels ≤500 mg/L, both with precipitants for the development of cast nephropathy; (4) dialysis independence within 12 months was associated with better OS; and (5) the serum FLC level at presentation was not associated with renal function recovery or with patient survival.

This study is the largest analysis to date of the relationship between FLC level in serum and biopsy-confirmed MCN. To the best of our knowledge only thirty-five studies have reported on serum FLC level in patients with biopsy-confirmed MCN^9,16–46^. The median numbers of patients reported in these studies were 5 (range 1–98) and the median serum FLC levels at diagnosis where available were: involved *κ* FLC 14,100 mg/L (range 80–42,000) and involved *λ* FLC 4267 mg/L (range 855–69,430).

Immunoassays for serum FLC have only entered widespread clinical practice in the last decade and there is limited data on the relationship between serum FLC level and MCN. The IMWG in their recent revised diagnostic criteria for MM included RI caused by MCN as a myeloma defining event, where MCN was characterised by the presence of typical histological features or an involved serum FLC level above 1500 mg/L^13^. The IKMG research group recommended an involved serum FLC level above 500 mg/L in patients with AKI as a threshold level for suspecting MCN and for consideration of an urgent haemato-oncological work up^14^. The results in the present study supports the IKMG threshold for MCN, as 5.8% of patients presented with a serum FLC level below 1500 mg/L; these patients would have been at risk of misdiagnosis if using the IMWG threshold for MCN. In a recent analysis of the myeloma IX clinical trial we found 22.7% of patients had a serum FLC level >1500 mg/L at presentation whereas 43.7% had serum FLC > 500 mg/L. In the same study, renal impairment defined as an eGFR <60 ml/min/1.73 m^2^ was noted in 71.4% of patients with serum FLC level above 1500 mg/L, and this was 60.6% in patients with serum FLC levels > 500 mg/L^15^. Importantly this study showed that patients could have a very high FLC level and no RI at diagnosis. This is consistent with experimental work which shows that each FLC has a specific level for precipitation. Animal studies also showed that the threshold for cast formation is lower in the presence of a co-precipitant^10^. The two patients with FLC levels ≤500 mg/L had co-precipitating factors.

There is great interest in improving the time to diagnosis and commencement of treatment for MCN. Hence, defining the serum FLC levels associated with MCN using the above threshold is an important component of achieving this aim. This would help by averting the need for delays and risks associated with renal biopsy, and consequent delay in initiation of life and organ saving anti-myeloma treatment. In addition, knowledge of early changes in serum FLC levels from baseline after commencement of chemotherapy in patients with MCN may also direct early changes in therapy. There is evidence that early disease response defined by a reduction in serum FLC is associated with an increased probability of renal recovery^47^.

A secondary aim of this study was to assess the association between serum FLC levels at presentation with renal recovery and OS in this population of patients who presented with severe RI. This analysis was limited by the change in time frame over which the study was performed and missing details of the chemotherapy regimens used for some patients. To date there are no studies that have reported on the relationship between serum FLC levels at diagnosis in patients presenting with MCN and subsequent renal recovery. We found that the level of serum FLC level at presentation had no influence on whether or not patients who required dialysis subsequently recovered independent renal function or remained dialysis-dependent.

There is however growing evidence that OS is improving substantially in patients with MM who require dialysis for AKI^48^; this improvement may be associated with increasing use of bortezomib-based regimens^49^. We did not have sufficient information on chemotherapy to perform an assessment of the impact of use of bortezomib, but by time-period we found a significantly better OS in patients who presented after 2008, the period when bortezomib came into widespread use for patients with MM and AKI, compared to patients who presented before 2008 (*P* = 0.006). Treatment of MM with bortezomib-based regimens became increasingly common after 2008 and the evidence in clinical and epidemiological studies for patients with MM and RI supports the benefits of this treatment^30,48^. The lack of association with extracorporeal removal of FLC is consistent with the major randomised controlled trials for plasma exchange and HCO-HD.in patients with MM and AKI^46,50,51^.

In conclusion, this is the largest study of the relationship between serum FLC levels and biopsy-confirmed MCN in patients with MM. 98% of patients with MCN at diagnosis presented with a serum FLC level > 500 mg/L. Based on the findings from this study we propose that a serum FLC level > 500 mg/L should be considered for future diagnostic criteria for MCN. This recommendation is only valid for the Freelite® FLC assay (The Binding Site, Birmingham) and validation with other FLC assays have not been performed. Serum FLC level at diagnosis was not associated with patient outcomes in MCN. Renal function recovery within 12 months was associated with improved survival in patients with MCN when compared to those who remained dialysis-dependent, no such benefit was observed after 12 months.
